# Challenges and caveats of a multi-center retrospective radiomics study: an example of early treatment response assessment for NSCLC patients using FDG-PET/CT radiomics

**DOI:** 10.1371/journal.pone.0217536

**Published:** 2019-06-03

**Authors:** Janna E. van Timmeren, Sara Carvalho, Ralph T. H. Leijenaar, Esther G. C. Troost, Wouter van Elmpt, Dirk de Ruysscher, Jean-Pierre Muratet, Fabrice Denis, Tanja Schimek-Jasch, Ursula Nestle, Arthur Jochems, Henry C. Woodruff, Cary Oberije, Philippe Lambin

**Affiliations:** 1 The D-Lab: Decision Support for Precision Medicine, GROW—School for Oncology and Developmental Biology, Maastricht University Medical Centre+, Maastricht, The Netherlands; 2 Department of Radiation Oncology (MAASTRO clinic), GROW—School for Oncology and Developmental Biology, Maastricht University Medical Centre+, Maastricht, The Netherlands; 3 OncoRay–National Center for Radiation Research in Oncology, Faculty of Medicine and University Hospital Cal Gustav Carus, Technische Universität Dresden, Helmholtz-Zentrum Dresden–Rossendorf, Dresden, Germany; 4 Department of Radiotherapy and Radiation Oncology, Faculty of Medicine and University Hospital Carl Gustav Carus, Technische Universität Dresden, Dresden, Germany; 5 Helmholtz-Zentrum Dresden–Rossendorf, Institute of Radiooncology—OncoRay, Dresden, Germany; 6 German Cancer Consortium (DKTK), Partner Site Dresden, and German Cancer Research Center (DKFZ), Heidelberg, Germany; 7 National Center for Tumor Diseases (NCT) Partner Site Dresden, Dresden, Germany; 8 German Cancer Research Center (DKFZ), Heidelberg, Germany; 9 Faculty of Medicine and University Hospital Carl Gustav Carus, Technische Universität Dresden, Dresden, Germany; 10 Helmholtz Association / Helmholtz-Zentrum Dresden–Rossendorf (HZDR), Dresden, Germany; 11 Centre Jean Bernard, Clinique Victor Hugo, Le Mans, France; 12 Department for Radiation Oncology, University Medical Center Freiburg, Freiburg, Germany; University of Chicago, UNITED STATES

## Abstract

**Background:**

Prognostic models based on individual patient characteristics can improve treatment decisions and outcome in the future. In many (radiomic) studies, small size and heterogeneity of datasets is a challenge that often limits performance and potential clinical applicability of these models. The current study is example of a retrospective multi-centric study with challenges and caveats. To highlight common issues and emphasize potential pitfalls, we aimed for an extensive analysis of these multi-center pre-treatment datasets, with an additional ^18^F-fluorodeoxyglucose (FDG) positron emission tomography/computed tomography (PET/CT) scan acquired during treatment.

**Methods:**

The dataset consisted of 138 stage II-IV non-small cell lung cancer (NSCLC) patients from four different cohorts acquired from three different institutes. The differences between the cohorts were compared in terms of clinical characteristics and using the so-called ‘cohort differences model’ approach. Moreover, the potential prognostic performances for overall survival of radiomic features extracted from CT or FDG-PET, or relative or absolute differences between the scans at the two time points, were assessed using the LASSO regression method. Furthermore, the performances of five different classifiers were evaluated for all image sets.

**Results:**

The individual cohorts substantially differed in terms of patient characteristics. Moreover, the cohort differences model indicated statistically significant differences between the cohorts. Neither LASSO nor any of the tested classifiers resulted in a clinical relevant prognostic model that could be validated on the available datasets.

**Conclusion:**

The results imply that the study might have been influenced by a limited sample size, heterogeneous patient characteristics, and inconsistent imaging parameters. No prognostic performance of FDG-PET or CT based radiomics models can be reported. This study highlights the necessity of extensive evaluations of cohorts and of validation datasets, especially in retrospective multi-centric datasets.

## Introduction

Prognostic models based on individual patient derived factors are essential to better estimate patient’s outcome prior to or during treatment. These models help to improve individualized treatment decisions (personalized medicine) that may lead to better patient outcomes [1, 2]. Medical images are an example of an information source, which could be used to derive important patient-specific prognostic information. Large amounts of quantitative features can be calculated from medical images acquired during a patient’s course of treatment, e.g. positron emission tomography (PET) or computed tomography (CT). This principle of extracting imaging features is called ‘radiomics’, which is a rapidly evolving field of interest [3–5]. Multiple studies have shown radiomics’ potential to derive prognostic information for patient outcomes. Despite the promising results, radiomics faces multiple challenges [6]. An important challenge is the collection and acquisition of (large amounts of) suitable imaging data, which is difficult due to evolving technology, lack of standardization protocols and differences in cohorts and protocols between institutes.

Imaging data collected from a single institution often results in a more homogeneous dataset, e.g. the images are acquired using the same settings (with the institute’s acquisition and reconstruction protocols) on the same scanner. Also, with regard to clinical characteristics the patient population is usually more homogeneous. However, a prognostic model developed based on these data might fail when applied to validation data from an external institute, due to the lack of transferability of the model (i.e. the model is specific for a population) [7, 8]. Therefore, one might argue for using more heterogeneous datasets to train the model to broaden the clinical applicability. In practice, it appears difficult to find these datasets and to validate such a model. Besides the variability in imaging and/or clinical characteristics within a dataset, the dimensionality of the data is often an issue, e.g. having over 1000 radiomic features with often only a limited number of patients. A review published in 2015 has shown that many published (radiomics) studies contain models based on a limited number of patients without validation on an independent dataset, resulting in high probabilities of false positive results [9].

In this study, PET and CT images were collected from non-small cell lung cancer (NSCLC) patients who underwent an ^18^F-fluorodeoxyglucose (FDG) positron emission tomography/computed tomography (PET/CT) scan prior to and during (chemo)radiotherapy to be able to evaluate radiomics’ potential to assess early response. Previous studies have explored (early) response assessment using information derived from FDG PET/CT scans [10–15]. Since the PET/CT scans obtained during treatment are generally not acquired in clinical practice, it is difficult to gather large datasets of this type. Therefore, the data in the current study were collected from three different institutes where small sub-cohorts were available and is therefore a typical example of a multi-centric study with retrospectively collected data.

Retrospective studies, although collected from centers with high quality care, often suffer from heterogeneities within and between datasets, which is unavoidable in clinical practice. Therefore, the current study aims to evaluate the multicenter patient data in terms of cohort characteristics and prognostic performance, intending to highlight common issues and emphasizing pitfalls for future (radiomic) studies.

## Materials and methods

### Study cohort

The entire study cohort consists of 138 stage II-IV NSCLC patients, all treated with curatively intended (chemo)radiotherapy. The study population was divided into four different datasets from three different institutes. Datasets 1 and 2 originated from MAASTRO Clinic, Maastricht, the Netherlands. Dataset 3 is from Clinique Victor Hugo, Le Mans, France, and Dataset 4 was obtained from University Medical Center Freiburg, Freiburg, Germany. Dataset 1 was prospectively collected with approval of the Institutional Review Board of the Department of Radiation Oncology of Maastricht University Medical Center (Maastro Clinic) (clinicaltrials.gov NTC00522639). The original study that collected the data in Dataset 2 was approved by the Institutional Board of the Department of Radiation Oncology of Maastricht University Medical Center (Maastro Clinic), as stated in [12]. Dataset 3 was collected in a previous study [16] and approved by the appropriate Institutional Review Board. Dataset 4 was prospectively collected with approval of the Ethics Committee of the Albert-Ludwig University Freiburg, Germany Freiburg (clinicaltrials.gov NCT00697333).

All patients underwent an ^18^F-FDG-PET/CT scan before radiotherapy for treatment planning purposes and an additional scan approximately two weeks after the start of treatment.

The scanning parameters are summarized in Table 1. Detailed cohort descriptions are provided in Supplementary Information: S1 Text.

**Table 1:** Scanning parameters. Scanning parameters for all scans included in the study.

Parameters		Dataset 1 (n = 100)	Dataset 2 (n = 62)	Dataset 3 (n = 54)	Dataset 4 (n = 60)
Manufacturer		Siemens	Siemens	Philips Healthcare	Philips Healthcare
CT	Tube voltage	120 kVp	120 kVp	120 kVp (n = 39)	120 kVp
				140 kVp (n = 15)	
	Tube current	120 mA (n = 3)	80 mA (n = 13)	Median [range]	Median [range]
		160 mA (n = 15)	336 mA (n = 49)	183 mA [115–277]	183 mA [35–337]
		173 mA (n = 1)			
		240 mA (n = 72)			
	Convolution kernel	B19f (n = 97)	B19f (n = 37)	B	B
		B30f (n = 2)	B30f (n = 12		
		B41f (n = 1)	B41f (n = 13)		
	Slice thickness	3 mm	3 mm	5 mm	1 mm (n = 16)
					2 mm (n = 16)
					3 mm (n = 4)
					4 mm (n = 24)
	Pixel spacing	0.98 mm	0.98 mm	1.2 mm (n = 44)	Median [range]
				1.4 mm (n = 10)	1.2 mm [0.7–1.4]
PET	Reconstruction algorithm	OSEM2D 4i8s	OSEM2D 4i8s	BLOB-OS-TF	BLOB-OS-TF
	Slice thickness	3 mm	3 mm	4 mm	2 mm (n = 38)
					4 mm (n = 22)
	Pixel spacing	4.07 mm	4.07 mm	4 mm	2 mm (n = 38)
					4 mm (n = 22)
	Injected FDG dose	Median [range]	Median [range]	Median [range]	Median [range]
		180 MBq [113–354]	302 MBq [175–482]	290 MBq [139–474]	340 MBq [198–434]
	Planned interval FDG injection–image acquisition	60 minutes	60 minutes	60 minutes	60 minutes

For all patients, overall survival (OS), calculated from the start of radiotherapy, was collected to serve as a clinical endpoint in this study. A patient still alive at the time of study analysis was considered right-censored. Median follow-up times were calculated using the Kaplan-Meier estimator with reversed events, considering death as censored data.

### Tumor segmentation and image analysis

The primary gross tumor volume (GTV) was delineated by experienced radiation oncologists on pre- and during-radiotherapy fused FDG-PET/CT images using the following WL settings, W:1700 L:-300 (lung) and for W:600 L:40 (mediastinum). These target delineations were not evaluated by other specialists and the reproducibility of these segmentations was not investigated in this study, as this segmentation process was just part of normal clinical practice. FDG-PET/CT images were converted into standard uptake value (SUV) prior to analysis [17]. A total of 1295 radiomic features were extracted from CT images and 1400 radiomic features were extracted from PET images. Prior to radiomics feature extraction, all images were resampled into voxel dimensions 1×1×3 mm^3^ for CT and 4×4×3 mm^3^ for PET, which corresponded to the average voxel dimensions of all images rounded to the nearest integer. Resampling decreases the variability of radiomic features [18] and was performed using cubic interpolation. For one PET scan the resampling resulted in a segment splitting off from the original volume resulting in two separate volumes. As radiomic features have a different interpretation when extracted from multiple volumes, this patient was excluded from the analyses to avoid inconsistencies.

The image analysis included the investigation of different feature groups: 1) Morphological ‘Shape’, 2) Fractal, 3) Local Intensity ‘LocInt’, 4) Statistical features ‘Stats’, 5) Intensity-volume histogram ‘IVH’, 6) Textural features, including gray-level co-occurrence matrix ‘GLCM’, gray-level run length matrix ‘GLRLM’, gray-level size zone matrix ‘GLSZM’, neighborhood gray tone difference matrix ‘NGTDM’, gray-level distance zone matrix ‘GLDZM’, neighborhood gray-level dependence matrix ‘NGLDM’, 7) Laplacian of Gaussian filter ‘LoG’ (prior to group 4), and 8) Wavelet filter (prior to group 6). The feature descriptions of the feature groups (fractal, local intensity and intensity histograms) can be found in the Supplementary Information S2 Text. Other feature descriptions can be found elsewhere [19]. The IVH features were extracted from PET images only and these allow to retrieve the metabolic tumor volume (MTV), which is defined as the volume of voxels with an intensity above x% of SUVmax within the lesion (from 10 to 90% (MTV_10%_—MTV_90%_)) [20] and total lesion glycolysis (TLG), by multiplying MTV by the corresponding mean SUV within the segmented volume: TLG_10%_—TLG_90%_ [20, 21].

Image analysis was performed in Matlab R2014a (The Mathworks, Natick, MA) using in-house developed software used for feature extraction. The absolute variation (abs) and percentage (rel) variation between subsequent scans were also derived, defined as:

$$Abs={During}_{treatment}-{Pre}_{treatment}$$ (1)

$$Rel=\frac{{During}_{treatment}-{Pre}_{treatment}}{{Pre}_{treatment}}$$ (2)

In total, eight sets of radiomic features were derived from each dataset, being CT-scan1 (pre-treatment), CT-scan2 (during treatment), PET-scan1, PET-scan2, CT-abs, CT-rel, PET-abs and PET-rel.

### Cohort comparison

To compare the four cohorts, two different approaches were applied. First of all, clinical characteristics were compared to test univariate cohort differences using a Wilcoxon rank test for continuous variables or a chi-square test for categorical variables. P-values below 0.05 were considered significant. Note that the p-values of these analyses were not corrected for multiple testing.

Secondly, the cohort differences (CD) model approach described in detail by van Soest *et al*. [7, 8] was used to assess the multivariate cohort differences by predicting to which cohort a patient belongs. It provides as summarizing measure of the level of generalizability of the model, ranging from reproducibility to transferability. Two CD-models were created. The first CD-model included two-year survival (binary variable) as an independent variable, as well as radiomic features of a model developed in the current study (see next section ‘Model development’). The second CD-model also included the clinical variables ‘Gender’ and ‘Overall Stage’ as independent variables to investigate whether potential differences between the cohorts can be explained by those clinical parameters. As two-year survival was used as input variable, four patients of Dataset 2 were not included in this analysis due to shorter follow-up times. For the CD-model, stage was converted into dummy variables, using three categories II, IIIa or IIIb/IV. Stage II was used as the reference category.

The binary dependent variable of the CD-models was cohort A or B. A simple logistic regression was used to train the regression beta coefficients and predict to which cohort the data belongs. This procedure was applied to all possible combinations of two datasets. The performance of the CD-models for each combination of two datasets was evaluated by their Receiver Operating Curve (ROC). High CD Area under the Curve (AUC) values would indicate a large difference between the distributions in the cohorts and imply that the model tests transferability rather than reproducibility [7, 8].

### Model development

Each of the four datasets was used to train a model, which was subsequently validated on the three remaining datasets. Moreover, a model was developed by combining all data into one large dataset (n = 138) which was split randomly into training (75%, n = 103) and validation (25%, n = 35), as proposed in [22].

For model development, a least absolute shrinkage and selection operator (LASSO) method was applied [23]. A 10-fold cross validation procedure was repeated 200 times to optimize the penalty coefficient lambda, i.e. to find the smallest error, and to stabilize the method. Inputs for LASSO were the total of extracted radiomic features from either CT-scan1, CT-scan2, PET-scan1, PET-scan2, CT-abs, CT-rel, PET-abs or PET-rel. The performance of the penalized Cox model was evaluated using Harrell’s concordance index (c-index), for which 1 indicates perfect discrimination and a value of 0.5 no discrimination (no greater than chance expectation) [24]. Moreover, prognostic index (PI) values, defined as ∑*_i_β_i_x_i_*, were calculated for all four datasets. These were analyzed to give insight in discriminative ability of the model, as proposed by Royston and Altman [25].

### Classification comparison

For the combined dataset, five different classifiers were investigated according to the methodology described by Deist, Dankers *et al*. [26]. These include glmnet (penalized generalized linear models), rf (random forest), svm (support vector machine), LogitBoost (boosting) and rpart (regression trees). The neural network classifier was not investigated, as these required too long computation times for a large number of input parameters and extensive tuning. The classifiers deal with binary outcomes, therefore ‘two-year survival’ was used as endpoint. Since four patients in Dataset 4 did not reach the minimum of two years of follow-up, these patients were excluded for this specific analysis. The maximum number of repetitions was increased to 47 (limited by available calculation time on the computer) for this study and default tuning was turned off. All other settings were kept to the standards as described in [26].

All statistical methods were performed in R (version 3.4.3), using the packages *survcomp*, *survival* and *glmnet*. For the classification comparison, additional packages were used as described in [26]. P-values below 0.05 were considered significant.

### Univariable analysis

The performance of commonly assessed PET metrics as potential prognostic factors was also investigated in an attempt to validate previous findings [10–16]. Also, radiomic features can be hard to interpret, whereas these PET metrics are well-known. A univariable Cox proportional hazard regression was computed for the percentage variation of most commonly assessed PET metrics: volume, maximum SUV (maximum image intensity value), mean SUV, peak SUV (maximum average SUV in a 1 cm^3^ spherical volume), MTV_50%_ (volume above 50% of intensity) and TLG_50%_ (TLG for the volume above 50% of intensity). This analysis was performed on the combined dataset of 138 patients.

## Results

Characteristics of the cohorts are summarized in Table 2, which shows that all clinical parameters were significantly different between one or more datasets, except age (indicated with the bold numbers). All patients from Datasets 1, 3 and 4 received concurrent chemoradiotherapy and no other treatment between the first PET scan and the start of radiotherapy (RT). In Dataset 2, 55% of patients received sequential chemoradiotherapy and one patient did not receive any chemotherapy. Because of the limited sample size available to us for this study, we have decided to not exclude outliers (e.g. PET scans separated by long time intervals) in an effort to make the dataset more homogeneous.

**Table 2:** Patient characteristics. Patient characteristics for all four datasets used in this study. The bold numbers 1, 2, 3 or 4 indicate the datasets from which the variable was significantly different.

	**Dataset 1 (n = 50)**	**Dataset 2 (n = 31)**	**Dataset 3 (n = 27)**	**Dataset 4** **(n = 30)**
**Age [years]**	**-**	**-**	**-**	**-**
Range (median)	35–86 (63)	46–82 (64)	41–76 (62)	47–83 (64)
Mean ± SD	62 ± 11	65 ± 9	61 ± 8	65 ± 9
**Gender**	**3**	**3**	**1,2,4**	**3**
Male	25 (50%)	22 (71%)	25 (93%)	19 (63%)
Female	25 (50%)	9 (29%)	2 (7%)	11 (37%)
**Stage**	**2,3,4**	**1**	**1**	**1**
II	-	2 (6%)	4 (15%)	1 (3%)
IIIa	17 (34%)	14 (45%)	16^a^ (59%)	16 (53%)
IIIb	27 (54%)	15 (48%)	7 (26%)	13 (43%)
IV	6 (12%)	-	-	-
**Histology**	**3,4**	**3,4**	**1,2**	**1,2**
Adenocarcinoma	18 (36%)	6 (19%)	11 (41%)	9 (30%)
Squamous cell carcinoma	14 (28%)	9 (29%)	14 (52%)	18 (60%)
NSCLC Otherwise Specified	18 (36%)	16 (52%)	2 (7%)	3 (10%)
**Radiotherapy [dose]**	**2,3**	**1,3,4**	**1,2**	**2**
Range (median)	45–69 (69)	46–70 (61)	66–70 (66)	30–74 (66)
Mean ± SD	64 ± 6	61 ± 7	68 ± 2	66 ± 8
**Chemotherapy**	**2**	**1,3,4**	**2**	**2**
Concurrent	50 (100%)	13 (42%)	27 (100%)	30 (100%)
Sequential	-	17 (55%)	-	-
No	-	1 (3%)	-	-
**Interval pre-PET–First RT [days]**	**3,4**	**3,4**	**1,2,4**	**1,2,3**
Range (median)	4–16 (7)	2–13 (7)	5–93 (33)	2–37 (16)
Mean ± SD	7 ± 2	8 ± 2	38 ± 21	17 ± 7
**Interval First RT–during-PET [days]**	**2,3**	**1,3,4**	**1,2,4**	**2,3**
Range (median)	5–20 (15)	6–19 (8)	15–32 (21)^b^	14–24 (15)
Mean ± SD	15 ± 2	9 ± 3	21 ± 4	16 ± 3
**Interval between PET scans [days]**	**2,3,4**	**1,3,4**	**1,2,4**	**1,2,3**
Range (median)	19–27 (22)	10–24 (16)	21–110 (52)	22–59 (33)
Mean ± SD	22 ± 2	17 ± 3	59 ± 21	34 ± 8

^a^Includes one TxN2M0 patient, for which the merged structure between node and tumor was analyzed.

^b^Two patients’ first PET scans were acquired more than 30 days after start of radiotherapy.

**Fig 1:**
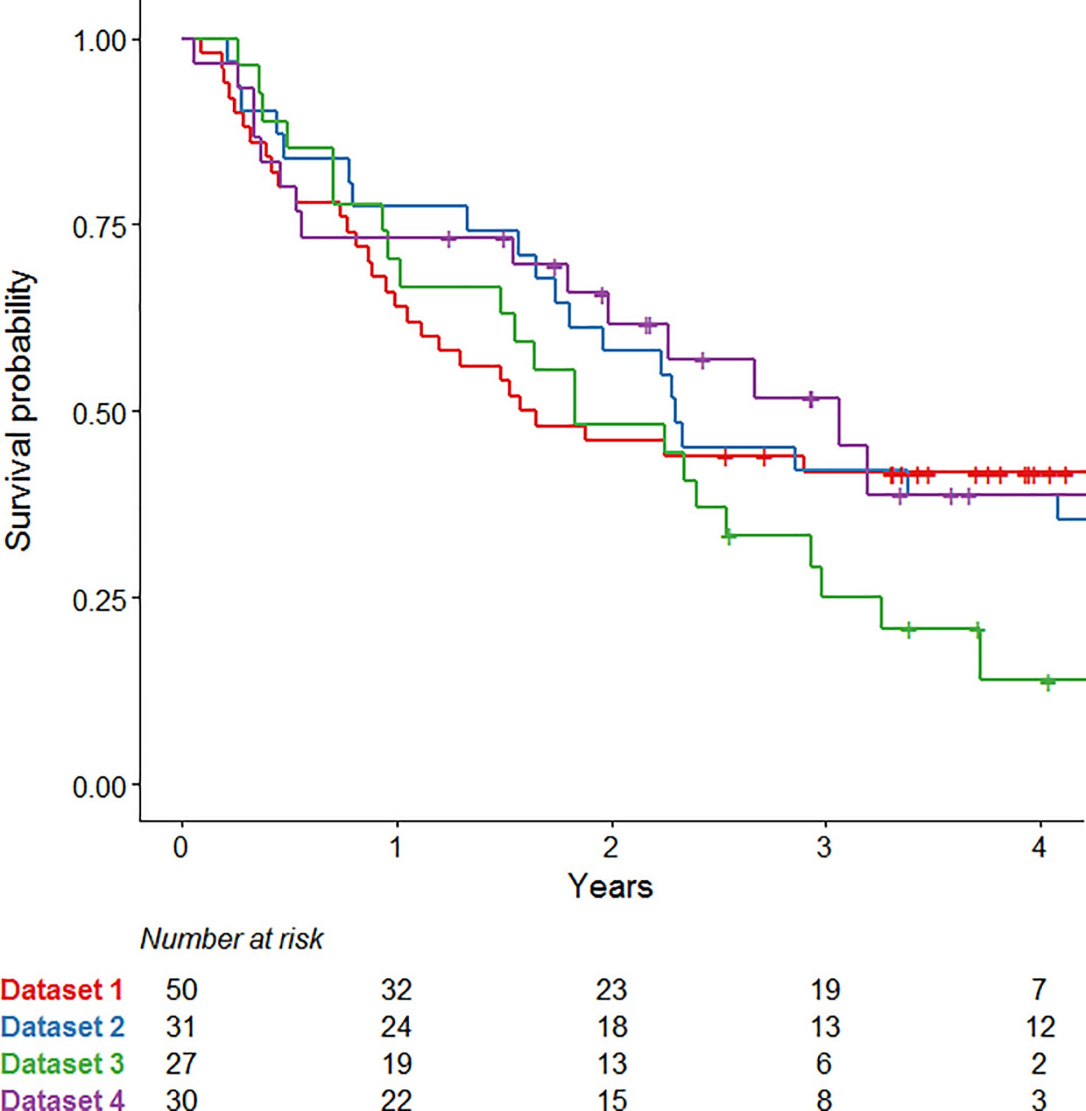
Kaplan-Meier curves. Kaplan-Meier curves for overall survival of all datasets.

Median [range] survival was 1.6 [0.1–4.5], 2.3 [0.2–7.0], 1.8 [0.3–5.9] and 3.1 [0.1–5.2] years for Dataset 1, 2, 3 and 4, respectively (Fig 1). Median follow-up was 3.8 [2.5–4.5], 6.9 [6.6–7.0], 4.0 [2.6–5.9] and 3.4 [1.3–5.2] years for Dataset 1, 2, 3 and 4, respectively.

**Fig 2:**
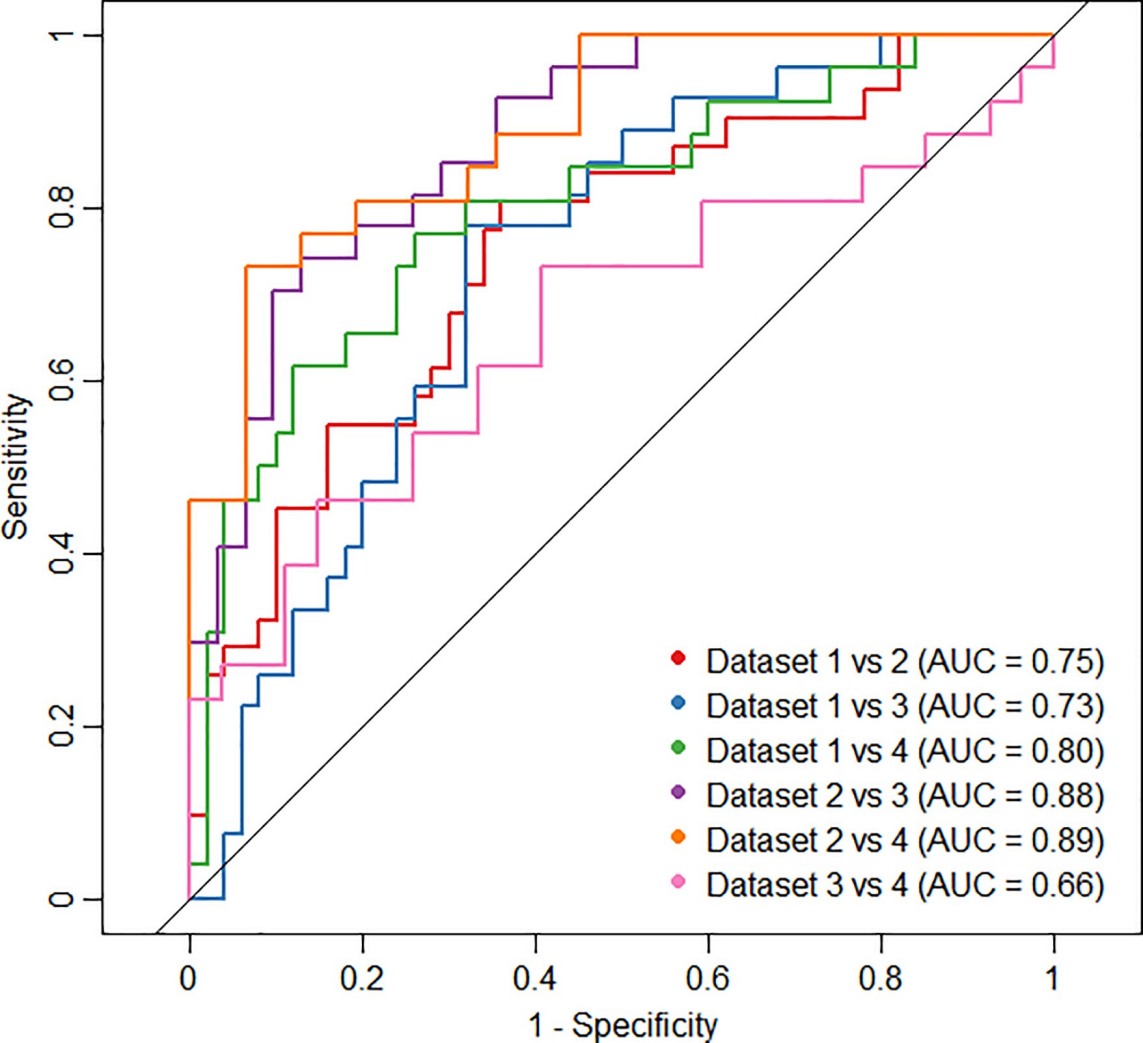
ROCs cohort difference model. Receiver Operator Curves (ROC) for the cohort difference (CD) model for each combination of datasets, including three radiomic features and ‘two-year survival’ as independent variables.

Fig 2 shows the ROCs of the CD-model for each combination of two datasets. AUC values ranged from 0.66 (Dataset 3 versus 4) to 0.89 (Dataset 2 versus 4). None of the confidence intervals included 0.5, meaning that all AUC values were significantly different from 0.5 (not shown). The ROC curves of the CD-model, which included gender and overall stage, resulted in slightly higher AUC values, ranging from 0.77 to 0.92 (S1 Fig).

**Fig 3:**
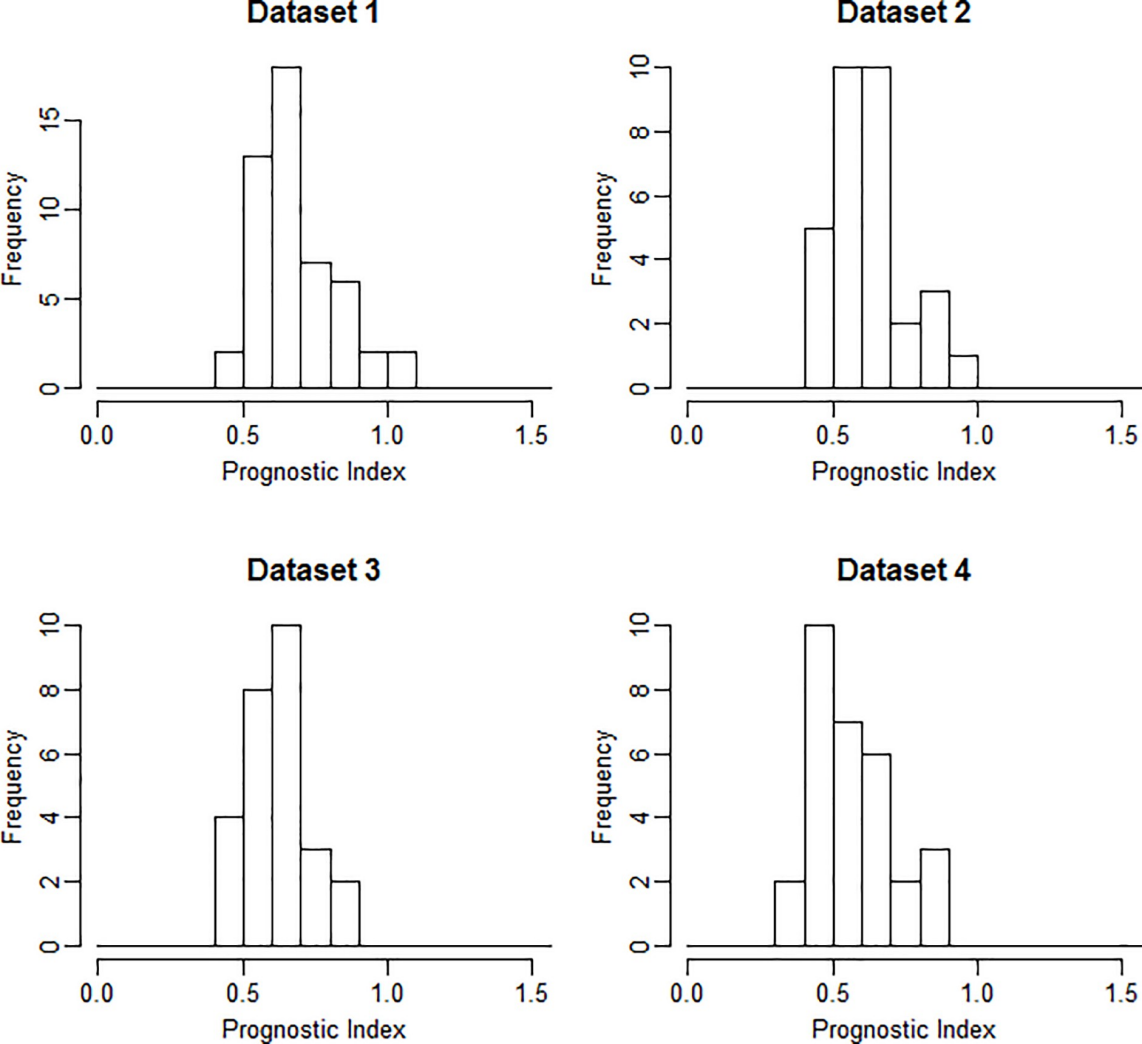
Prognostic index. Prognostic index (PI) ranges for all datasets based on the model developed on CT–scan1, using Dataset 1 as training dataset.

To assess prognostic performance, models were developed using all four different datasets once as training and the remainders as validation. The first models were trained on the image sets of the largest dataset, Dataset 1 (n = 50). Using LASSO, prognostic predictors were identified for the image sets CT-scan1, CT-rel and PET-abs. For the other image sets, no prognostic models were identified. Since Dataset 1 was the largest dataset and CT scans prior to treatment are currently most commonly used to assess prognostic performance, the model belonging to CT-scan1 of Dataset 1 was chosen to be used to construct the CD-model (Fig 2). This model, built using LASSO, consisted of 3 variables: 1) Wavelet LLH (Low Low High) Fractal sd (standard deviation), 2) Wavelet LLH GLDZM IV (Inverse Variance) and 3) Wavelet LLH GLSZM IV. The corresponding beta coefficients of the Cox model were 1.38, 5.0*10^−17^ and 2.9*10^−32^, respectively. Fig 3 shows the ranges of the prognostic index (PI) for all four datasets for this model.

In Table 3 shows the results for Harrell’s concordance index for the imaging features extracted from all image sets and the calculated differences. The performances of models trained on Dataset 2, 3 or 4 are shown in Supplementary Information S3 Text.

**Table 3:** Model performance. Values of Harrell’s concordance index with 95% confidence intervals, in the case Dataset 1 (n = 50) was used as training (T) to develop a model using LASSO. Validation (V) results are shown for Dataset 2, 3 and 4. Significant values are indicated in grey. A hyphen indicates that either all coefficients were forced to zero, or all predictions were equal to one, meaning that no linear combination of any subset of regressors was useful in predicting the outcomes.

	CT-scan1	CT-scan2	PT-scan1	PT-scan2	CT-rel	CT-abs	PT-rel	PT-abs
**T-Dataset 1**	**0.68**	**-**	**-**	**-**	**0.67**		**-**	**0.64**
T-Lower bound	0.59	-	-	-	0.57		-	0.56
T-Upper bound	0.76	-	-	-	0.76		-	0.73
**V-Dataset 2**	**0.55**	**-**	**-**	**-**	**0.58**		**-**	**0.56**
V-Lower bound	0.40	-	-	-	0.48		-	0.43
V-Upper bound	0.69	-	-	-	0.69		-	0.70
**V-Dataset 3**	**0.41**	**-**	**-**	**-**	**0.53**		**-**	**0.56**
V-Lower bound	0.27	-	-	-	0.39		-	0.44
V-Upper bound	0.54	-	-	-	0.68		-	0.68
**V-Dataset 4**	**0.49**	**-**	**-**	**-**	**0.62**		**-**	**0.52**
V-Lower bound	0.37	-	-	-	0.48		-	0.36
V-Upper bound	0.62	-	-	-	0.75		-	0.68

Furthermore, all data was combined into one large cohort and randomly split into training (n = 103) and validation (n = 35). For CT-rel and CT-abs, models were selected with c-indices significantly different from 0.5: 0.68 [95% C.I. 0.61–0.74] and 0.68 [95% C.I. 0.61–0.74], respectively. However, none of these models could be validated on the validation subgroup, reaching c-indices of 0.54 [95% C.I. 0.42–0.67] and 0.50 [95% C.I. 0.37–0.62]. For the other image sets, subsets of regressors were identified which resulted in predictions (i.e. relative risks) equal to 1 for all patients, meaning that the model did not contain any prognostic information.

**Fig 4:**
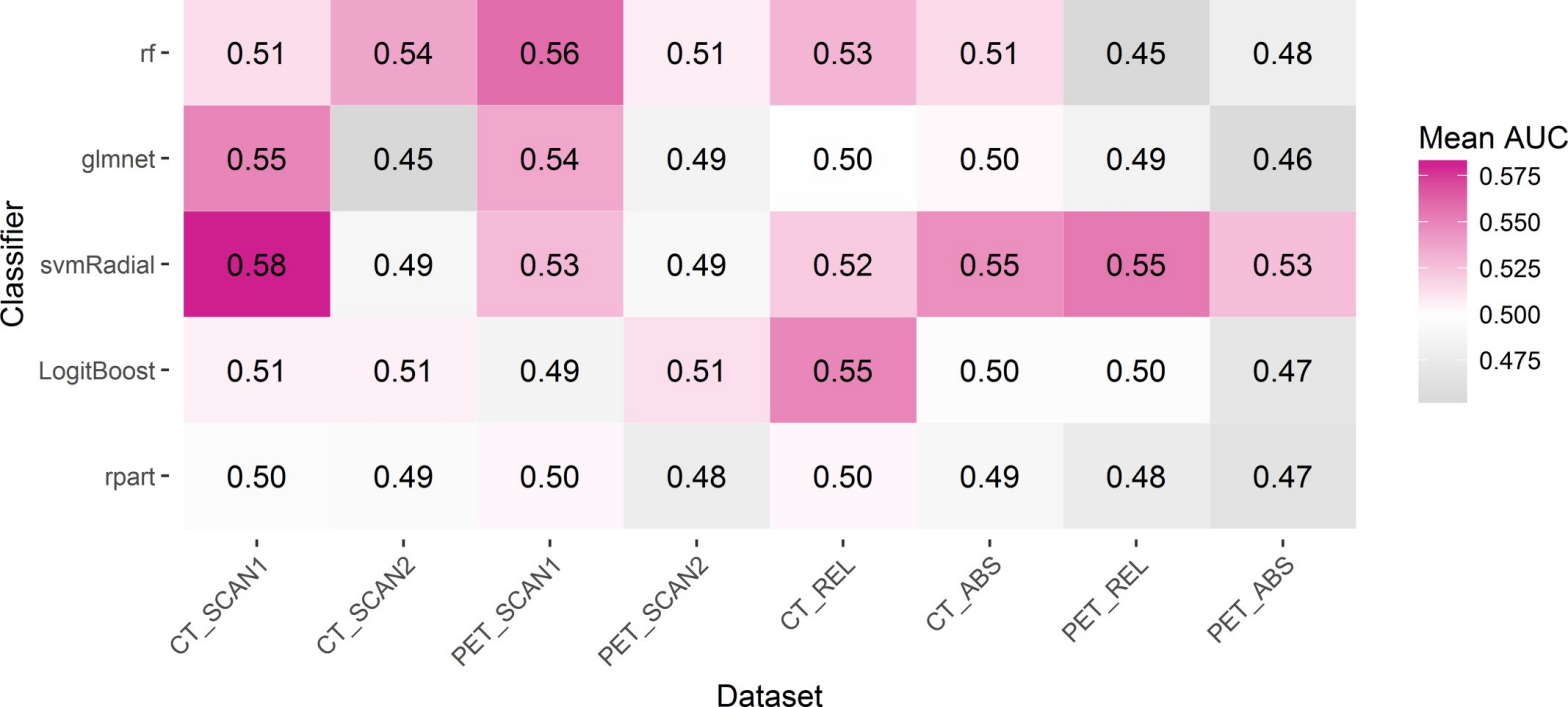
Classifiers heatmap. Heatmap with the mean AUC values found for each classifier and each image set, predicting two-year survival. The entire cohort with at least two-years of follow-up (n = 134) was used in this investigation.

Besides LASSO, different classifiers were investigated to further examine the prognostic value of the radiomic features for 2-year survival. The mean AUC values are shown in the heatmap of Fig 4.

Table 4 shows the results of the univariable analysis of the prognostic value of percentage variation of common PET imaging descriptors on the combined cohort of 138 patients. The results for hazard ratio (HR), corresponding confidence interval (CI) and the c-index were shown.

**Table 4:** Univariable analysis. Cox regression on the percentage variation of the PET imaging descriptors most commonly used, reporting the univariable hazard ratio (HR), 95% confidence interval (CI) of the HR and corresponding p-value. Univariable performance is reported in terms of the concordance-index (c-index). Absolute values of Scan 1 and 2, and percentage variation between PET acquisitions of the analyzed metrics are also presented (mean ± standard deviation).

	Scan 1	Scan 2	Percentage difference	HR	95% CI	p-value	c-index
Volume [cm^3^]	85 ± 113	68 ± 94	-20 ± 31	1.11	0.55–2.25	0.77	0.50
SUV max	12 ± 6.2	9.0 ± 4.1	-20 ± 39	1.16	0.73–1.83	0.53	0.52
SUV mean	5.3 ± 2.5	4.1 ± 1.8	-12 ± 71	1.09	0.87–1.36	0.45	0.55
SUV peak	8.8 ± 4.3	6.4 ± 2.8	-18 ± 51	1.14	0.83–1.56	0.43	0.54
MTV 50%	26 ± 39	20 ± 25	-9.5 ± 46	1.72	1.09–2.71	0.02	0.54
TLG 50%	266 ± 499	132 ± 212	-25 ± 61	1.19	0.92–1.54	0.18	0.56

## Discussion

This study aimed to show potential issues using a typical problematic example of retrospective multi-centric study, by investigating different methodologies and approaches for comparing cohort characteristics and assessing prognostic performance in a radiomic study. The analysis was based on a combined evaluation of four sub-cohorts, which resulted in the inclusion of 138 patients. The acquisition of a larger dataset is challenging for this research question, as FDG-PET/CT scans during treatment are not standard in clinical routine. As far as we are aware, the current dataset is one of the largest available. Nevertheless, the amount of data is probably less than required in order to build a generalized feature model for repeated PET/CT studies. Although the size of this dataset may not be sufficient from a statistical point of view, its results may provide clinical relevant insights to be obtained in order to improve future studies with limited sample sizes. Therefore, we think it is important to publish these results in an attempt to guide reflections and considerations with respect to analyses and conclusions in future (radiomic) studies. In this study, both the characteristics of the study cohort and the prognostic performance of imaging features were extensively explored.

First of all, comparison of the cohort characteristics of all datasets showed that all variables were significantly different between two or more datasets, except for age. The overall stage, as well as the received radiotherapy dose of the patients in Dataset 1, was significantly different compared to the other three datasets. In addition, the timing of chemotherapy was different, since 55% of the patients from Dataset 2 received sequential chemotherapy, whereas all other patients received concurrent chemotherapy. Furthermore, the intervals between the two FDG-PET/CT scans was quite different between the datasets, since this was a retrospective study and the initial purposes of the studies were different. The variations in treatment types as well as differing outcomes between the cohorts are a probable source of noise that dilutes any signal present within the radiomic features. Furthermore, heterogeneities introduced by differences in timing of the imaging sessions, e.g. between PET scans, are a well-known confounding factor in retrospective imaging studies. Besides the large variabilities of clinical variables between the datasets, the CD-model showed high AUC values (0.65–0.88). The high AUC values of the CD-model indicate high ability to predict to which dataset the data belongs. In other words, high AUC values mean that the (distribution of) the input variables of the cohort are very different. This may be caused by differences in radiomic features since the survival is very similar for all datasets (Fig 1). Moreover, this shows that the model tests the transferability rather than the reproducibility of the model, i.e. the model is specific for a population. While the exclusion of patients with characteristics considered to be outliers would have resulted in a more homogeneous dataset, we decided against this approach as it would have resulted in too small a dataset to draw any conclusions from.

Secondly, the prognostic performance of the radiomic features extracted from CT, PET, delta-CT or delta-PET, was investigated. The models were selected using a LASSO procedure. High values of Harrell’s concordance index (around 0.7 and even reaching 0.86) indicate good prognostic performance of the models trained on Dataset 2. Nevertheless, the results could not be validated on independent datasets, with the only significant c-index being 0.64 achieved on Dataset 4 for PET-scan1. This c-index was the only value being significantly different from 0.5. When one of the other datasets was used as training, no model achieved a c-index significantly different from 0.5 on any of the remaining validation datasets. In terms of discriminative power of the model, the spread of prognostic indices shown for the model trained on CT-scan1 of Dataset 1 does not show relevant outliers or differences between the datasets. In summary, the model performance results show the importance of validation: one should be careful with presenting results that are developed on (small) datasets without validation, as they are likely to be over-optimistic. In case only one (large) dataset is available, a cross-validation procedure could be applied to reduce the risk of overfitting. A recent paper summarizes the need for validation to assess the clinical usefulness of prognostic models [27].

Potentially, LASSO (e.g. penalized regression) was not the optimal method to select a prognostic model. Therefore, multiple other classifiers [28] were investigated using the methodology described by Deist *et al*. [26]. Since the sub-cohorts were small, all data were combined into one large cohort to investigate the ability of five different classifiers to predict 2-year survival. None of the classification methods was able to produce a clinical significant result, with the highest mean AUC being 0.58 for the radiomic features extracted from CT images prior to treatment. This result was achieved by the support vector machine (svm), which overall obtained the highest AUC results. Combining datasets into one large cohort did also not result in significant c-indices, potentially influenced by the heterogeneity within the combined dataset, which makes it rather difficult to develop a prognostic model.

The performance of the percentage variation of commonly used PET metrics was also investigated. Only MTV_50%_ achieved a significant Hazard Ratio, but the corresponding c-index of the Cox model was not significantly different from 0.5. This performance was also performed on the PET features extracted from either scan 1 or 2 instead of the percentage variation. None of the metrics was significantly related to overall survival (results not shown). This contradicts previously published results, which have shown the importance of these metrics in a prognostic setting [29, 30].

The results do not show conclusive results for the possibility to perform early treatment response assessment using an additional ^18^F-FDG-PET/CT scan acquired at intermediate time point during treatment with a radiomics analysis. However, the potential prognostic value of differences between two time points was investigated before and showed promising, but also conflicting results [10, 12, 14, 16, 31]. Cremonesi *et al*. [32] reports that the differences in PET parameters are a main limitation for 21 recent studies in early response assessment for NSCLC patients. In general, radiomic features are highly affected by different acquisition and reconstruction settings [33–39]. This is one of the factors that could (partly) explain the results in the current study. In this study, there were large differences between datasets, but even within a dataset the acquisition and reconstruction settings were not identical, as shown in Table 1. The inconsistency of settings within a dataset is an issue which is difficult to overcome, especially in retrospective studies. Initiatives to provide protocols and guidelines will hopefully improve standardization in the future [40–42]. Moreover, a recent study proposed a ‘post-reconstruction harmonization method’ to reduce the variability in radiomic features extracted from PET images from different institutes [43]. Besides the inconsistency in acquisition and reconstruction settings within a dataset, other parts of the radiomics workflow including segmentation [44], pre-processing [45, 46] and feature extraction, remain problematic for multi-center studies and the transferability and reproducibility of developed models. A recent review proposed a harmonization for the radiomics methodology [47]. Moreover, the image biomarker standardization initiative (ISBI) attempts to standardize radiomics in terms of feature definitions and processing [48]. Nevertheless, the lack of standardization remains one the main limitations for radiomic studies. Several factors can improve the quality of future radiomic studies and reduce the risk of false positive results, as summarized in the recently proposed Radiomics Quality Score (RQS) [3].

The descriptive character of this study is intended to serve as a tool for highlighting common issues in radiomics literature and to emphasize pitfalls for future studies. Moreover, we emphasize the urgent need to publish negative results to avoid publication bias. With the current study, we would like to highlight the importance of proper validation of the results, but also the consideration of the feasibility of performing a (radiomic) study. We would recommend to perform statistically rigorous sample size calculations upfront and to perform an extensive cohort investigation to decrease the risk of false positive findings. It is essential to reduce this risk, as the main goal is to improve individual treatment decisions for better patient outcome, which can only be achieved when proper statistically sound investigations are reported.

## Conclusion

In an attempt to inform future radiomic studies, we illustrate possible problems that can be encountered in retrospective multi-centric study by evaluating cohort characteristics and clinical characteristics. The models presented do not support any correlation between radiomic features acquired from FDG-PET/CT scans and overall survival. Further investigations indicate that the radiomic analysis was influenced by the limited sample size and heterogeneous imaging and clinical characteristics.

## Supporting information

S1 Text Dataset descriptions. (DOCX)

S1 Fig ROCs cohort difference model. Receiver Operator Curves (ROC) for the cohort difference (CD) model for each combination of datasets, including three radiomic features, ‘two-year survival’, ‘gender’ and ‘stage’ as independent variables.
(TIFF)

S2 Text Feature descriptions. Mathematical feature definitions of the feature groups ‘Fractal’, ‘Local Intensity’ and ‘Intensity Histogram’.
(DOCX)

S3 Text Model performance. Harrell’s concordance index with 95% confidence intervals for models trained on Dataset 2, 3, or 4 and validated on the remaining datasets.
(DOCX)
