# Supplementary material for: Challenges and caveats of a multi-center retrospective radiomics study: an example of early treatment response assessment for NSCLC patients using FDG-PET/CT radiomics
Source: PLoS One. 2019 Jun 3;14(6):e0217536. doi: 10.1371/journal.pone.0217536 (PMC6546238; doi:10.1371/journal.pone.0217536)
Supplement: S1 Text — (DOCX) [file pone.0217536.s001.docx]

**Dataset Descriptions**

Dataset 1

The first dataset consists of 50 stage IIIa-IV NSCLC patients treated between September 2010 and March 2012. Data was prospectively collected (NTC00522639). All patients have given written informed consent. Patients with prior history of lung cancer or patients undergoing surgery were excluded. All patients were treated with curatively intended (chemo)radiotherapy. A larger cohort of patients from the same clinical trial were studied in [1], but use in the current study was limited due to availability of during treatment FDG-PET/CT images.

Before scanning, patients fasted for a minimum of 6 hours. The total administered dose of FDG was calculated as (bodyweight (Kg) x 4 + 20) MBq. FDG-PET/CT images were acquired 60 minutes post injection on a Siemens Biograph 40 Truepoint PET/CT (Siemens AG, Munich, Germany). An Ordered Subset Expectation Maximisation 2D 4 iterations 8 subsets (OSEM2D 4i8s) algorithm with a 5 mm post-reconstruction Gaussian smoothing filter was used for PET image reconstruction. Voxel size was 4.07 x 4.07 x 3.0 mm^3^. All PET scans were corrected for attenuation using the mid-ventilation phase of the 4DCT or a 3DCT thorax, case the 4DCT was not of sufficient image quality due to irregular breathing of the patient. Model-based methods were applied for scatter correction, and all scans were corrected for random events and decay. All CT images were acquired using a peak tube voltage of 120 kVp and a tube current of 120 mA, 160 mA, 173 mA or 240 mA. All images were reconstructed to a voxel size of 0.977 x 0.977 x 3.0 mm^3^ using the kernel B19f in 97% of the cases.

Dataset 2

The second dataset comprised 32 stage IIb-IIIb NSCLC patients, originating from a dataset described previously by van Elmpt et al. [2]. Patients were treated between July 2008 and December 2008. The same procedure for PET imaging and the same imaging protocol and settings were used to acquire the PET/CT images as described for dataset 1. The tube currents used were 80 mA and 336 mA and the convolution kernels were B19f, B30f and B40f.

Dataset 3

Dataset 3 consists of 27 stage II-IIIb NSCLC patients. Characteristics of this dataset has been described before [3]. Patients were treated between June 2008 and December 2012. FDG-PET/CT images were acquired 60 minutes post injection on a Philips Gemini TF 64 PET/CT System (Philips Healthcare, Cleveland, OH). The second PET/CT scan was acquired after 30 Gy was given. PET images were reconstructed to isotropic voxels of 4.0 x 4.0 x 4.0 mm^3^ using the default algorithm BLOB Ordered Subset Time of Flight (BLOB-OS-TF). CT images were acquired using a peak tube voltage of 120 or 140 kVp and variable tube currents ranging between 115 and 277 mA. The images were reconstructed to a pixel size of 1.17 x 1.17 mm^2^ or 1.37 x 1.37 mm^2^ and a slice thickness of 5.0 mm using kernel B.

Dataset 4

The fourth dataset consists of 30 stage II-IIIb NSCLC patients, recruited in a subproject of a multicenter PET-PLAN trial (drks.de DRKS00002178). Patients were treated between August 2010 and July 2015. Inclusion and exclusion criteria were according to the main study ‘PET-PLAN’. All patients received concurrent chemotherapy, either Cisplatin or Carboplatin on day 1 and 5 of week 1 and 5 of radiotherapy and Vinorelbine (Navelbine) on day 1 of week 1, 2, 3, 5, 6 and eventually week 7. The second PET/CT was made after between 16 and 30 Gy was given (median ± sd = 22 ± 3). PET/CT images were acquired on a Philips Gemini TF 64 PET/CT System or the Philips Gemini TF Big Bore PET/CT (Philips Healthcare, Cleveland, OH). PET images were reconstructed using the default algorithm (BLOB-OS-TF). All images were attenuation corrected and corrected for random events, scatter and decay. Except for one scan (5.35 x 5.35 x 3.0 mm), all PET images were reconstructed into a voxel size of 4.07 x 4.07 x 3.0 mm^3^. CT images were acquired using a peak tube voltage of 120 kVp and a tube current ranging between 35 mA and 337 mA. CT pixels sizes range between 0.684 x 0.684 mm^2^ and 1.37 x 1.37 mm^2^, and slice thickness between 1 mm and 5 mm. Kernel B was used.

**References**

1. Carvalho S, Leijenaar RTH, Troost EGC, van Timmeren JE, Oberije C, van Elmpt W, et al. 18F-fluorodeoxyglucose positron-emission tomography (FDG-PET)-Radiomics of metastatic lymph nodes and primary tumor in non-small cell lung cancer (NSCLC) - A prospective externally validated study. PloS one. 2018;13(3):e0192859. Epub 2018/03/02. doi: 10.1371/journal.pone.0192859. PubMed PMID: 29494598; PubMed Central PMCID: PMCPMC5832210.

2. van Elmpt W, Ollers M, Dingemans AM, Lambin P, De Ruysscher D. Response assessment using 18F-FDG PET early in the course of radiotherapy correlates with survival in advanced-stage non-small cell lung cancer. Journal of nuclear medicine : official publication, Society of Nuclear Medicine. 2012;53(10):1514-20. Epub 2012/08/11. doi: 10.2967/jnumed.111.102566. PubMed PMID: 22879081; PubMed Central PMCID: PMCPMC4667805.

3. Yossi S, Krhili S, Muratet JP, Septans AL, Campion L, Denis F. Early assessment of metabolic response by 18F-FDG PET during concomitant radiochemotherapy of non-small cell lung carcinoma is associated with survival: a retrospective single-center study. Clinical nuclear medicine. 2015;40(4):e215-21. Epub 2014/12/30. doi: 10.1097/rlu.0000000000000615. PubMed PMID: 25546211.
