# Supplementary figures and images for: Challenges and caveats of a multi-center retrospective radiomics study: an example of early treatment response assessment for NSCLC patients using FDG-PET/CT radiomics
Source: PLoS One. 2019 Jun 3;14(6):e0217536. doi: 10.1371/journal.pone.0217536 (PMC6546238; doi:10.1371/journal.pone.0217536)

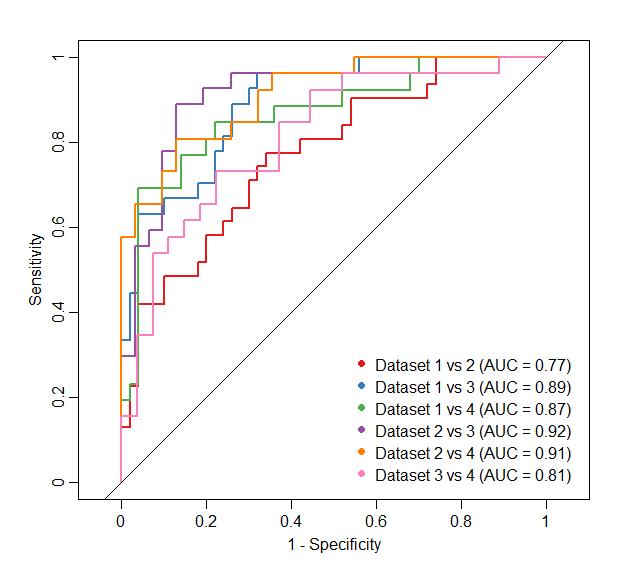

Supplement: S1 Fig — Receiver Operator Curves (ROC) for the cohort difference (CD) model for each combination of datasets, including three radiomic features, ‘two-year survival’, ‘gender’ and ‘stage’ as independent variables. (TIFF) [file pone.0217536.s002.tiff]
