# Supplementary material for: Challenges and caveats of a multi-center retrospective radiomics study: an example of early treatment response assessment for NSCLC patients using FDG-PET/CT radiomics
Source: PLoS One. 2019 Jun 3;14(6):e0217536. doi: 10.1371/journal.pone.0217536 (PMC6546238; doi:10.1371/journal.pone.0217536)
Supplement: S2 Text — Mathematical feature definitions of the feature groups ‘Fractal’, ‘Local Intensity’ and ‘Intensity Histogram’. (DOCX) [file pone.0217536.s003.docx]

**Feature descriptions**

***Fractal Dimension features***

The Fractal Dimension (FD) of the image is computed as described by Al-Kadi and Watson (1). Given the FD processed image $I$, with $N$ elements:

1. **Average:**

$$average=\frac{1}{N}\sum I$$

2. **Lacunarity**

$$lacunarity=\frac{\frac{1}{N}\sum I^{2}}{\frac{1}{N^{2}}\sum I}-1$$

3. **Standard deviation:**

$$standard deviation=\left( \frac{1}{N-1}\sum{(I-\overline{I})}^{2} \right)^{1/2}$$

Where $\overline{I}$ is the mean of $I$.

***Local Intensity features***

Local Intensity (LocInt) features are defined based on local intensity values around a center voxel (2).

1. **Local intensity peak**

Mean intensity level in a 1 cm3 spherical volume, centered on the voxel with the maximum intensity level in the volume of interest. In case multiple voxels contain the maximum intensity level, the highest mean intensity level of all spherical volumes is used.

1. **Global Intensity peak**

Similar to local intensity peak, but in this case the mean intensity level in a 1 cm3 ­­spherical volume is calculated for every voxel in the volume of interest. The highest mean intensity level of all spherical volumes is selected as the global intensity peak feature.

***Intensity histogram features***

Intensity histogram features describe the distribution of grey values within the volume, after discretization into intensity level bins was applied. Let:

$X_{d}=\{X_{d,1},X_{d,2},\ldots,X_{d,N_{v}}\}$ be the set of discretized intensity values of the $N_{v}$ voxels in the volume of interest,

$H=\{n_{1},n_{2},\ldots\}$ be the histogram with frequency count $n_{i}$ of each discretized intensity level $i$ in $X_{d}$,

$N_{g}$ be the number of discretized intensity values (bins) in the volume of interest,

$p_{i}$ be the occurrence probability for each bin $i$ of the histogram $N_{g}$, where $p_{i}=n_{i}/N_{v}$.

1. **Coefficient of variance (cov)**

$$cov= \frac{standard deviation}{\mathrm{mean}}$$

2. **Energy** $energy=\sum_{j=1}^{N_{v}} {X_{d}(j)}^{2}$

Energy is also known as the sum of squares.

1. **Entropy**

$$entropy=- \sum_{i=1}^{N_{g}} P(i)\log_{2} P(i)$$

2. **Interquartile range (iqr)**

$$IQR=P_{75}-P_{25}$$

where $P_{25}$ and $P_{75}$ are the 25th and 75th percentile of $X_{d}$, respectively.

1. **Kurtosis**

$$kurtosis=\frac{\frac{1}{N_{v}}\sum_{j=1}^{N_{v}} {(X_{d}\left( j \right)-\bar{X}_{d})}^{4}}{\left( \frac{1}{N_{v}}\sum_{j=1}^{N_{v}} \left( X_{d}\left( j \right)-\bar{X}_{d} \right)^{2} \right)^{2}}$$

where $\bar{X}_{d}$ is the mean of $X_{d}$.

1. **Maximum**

The maximum discretized intensity value of $X_{d}$.

$$maximum=\max(X_{d})$$

1. **Maximum histogram gradient (maxgrad)**

$$maxgrad=\max(H^{'})$$

Where $H'$ is the histogram gradient, defined as:

$$H^{'}=\{H\left( 2 \right)-H\left( 1 \right),\ldots,\frac{H\left( i+1 \right)-H\left( i-1 \right)}{2},\ldots,H\left( N_{g} \right)-H(N_{g}-1)\}$$

1. **Maximum histogram gradient intensity level (maxgradi)**

The discretized intensity level $i$ corresponding to the maximum histogram gradient.

1. **Mean**

The mean discretized intensity value of $X_{d}$.

$$mean= \frac{1}{N_{v}}\sum_{j=1}^{N_{v}} X_{d}(j)$$

1. **Mean absolute deviation (meand)**

The mean of the absolute deviations of all discretized intensity levels around the mean of $X_{d}$.

$$meand= \frac{1}{N_{v}}\sum_{j=1}^{N_{v}} \left| X_{d}\left( j \right)-\bar{X_{d}} \right|$$

where $\bar{X}_{d}$ is the mean of $X_{d}$.

1. **Median**

The sample median of $X_{d}$ or the 50th percentile of $X_{d}$.

1. **Median absolute deviation (mediand)**

The dispersion from the median of $X_{d}$.

$$mediand= \frac{1}{N_{v}}\sum_{j=1}^{N_{v}} \left| X_{d}\left( j \right)-M \right|$$

where $M$ is the median of $X_{d}$.

1. **Minimum**

The minimum discretized intensity value of $X_{d}$.

$$minimum=\min(X_{d})$$

1. **Minimum histogram gradient (mingrad)**

$$mingrad=\min(H^{'})$$

Where $H'$ is the histogram gradient, defined as:

$$H'=\{H\left( 2 \right)-H\left( 1 \right),\ldots,\frac{H\left( i+1 \right)-H\left( i-1 \right)}{2},\ldots,H\left( N_{g} \right)-H(N_{g}-1)\}$$

1. **Minimum histogram gradient intensity level (mingradi)**

The discretized intensity level $i$ corresponding to the minimum histogram gradient.

1. **Mode**

The mode of $X_{d}$ is the most frequently occurring discretized image level present. In case multiple bins have the highest count $n_{i}$, the mode is the smallest of those values.

1. **Uniformity**

$$uniformity= \sum_{i=1}^{N_{g}} {P(i)}^{2}$$

2. **Range**

The range of bins in the histogram, i.e. the width of the histogram.

$$range=\max\left( X_{d} \right)-min(X_{d})$$

1. **Root mean square (RMS):** $RMS=\sqrt{\frac{\sum_{j=1}^{N_{v}} {X_{d}(j)}^{2}}{N_{v}}}$
2. **Robust mean absolute deviation (rmeand)**

Similar to mean absolute deviation, but in this case only considering the set of intensity levels in the range between the 10th and 90th percentile of $X_{d}$.

$$rmeand= \frac{1}{N_{10-90}}\sum_{j=1}^{N_{10-90}} \left| X_{d,10-90}\left( j \right)-\bar{X}_{d,10-90} \right|$$

where $X_{10-90}$ represents the set of $N_{10-90}$ voxels in $X_{d}$ whose discretized intensity levels fall within the range of the 10th till the 90th percentile of $X_{d}$.

1. **Skewness**

$$skewness=\frac{\frac{1}{N_{v}}\sum_{j=1}^{N_{v}} \left( X_{d}\left( j \right)-\bar{X}_{d} \right)^{3}}{\left( \sqrt{\frac{1}{N_{v}}\sum_{j=1}^{N_{v}} \left( X_{d}\left( j \right)-\bar{X}_{d} \right)^{2}} \right)^{3}}$$

2. **Standard deviation**

$$standard deviation=\left( \frac{1}{N_{v}-1}\sum_{j=1}^{N_{v}} {(X_{d}\left( j \right)-\bar{X}_{d})}^{2} \right)^{1/2}$$

3. **Variance**

The variance of $X_{d}$.

$$variance= \frac{1}{N_{v}-1}\sum_{j=1}^{N_{v}} {{(X}_{d}\left( j \right)-\bar{X_{d}})}^{2}$$

where $\bar{X_{d}}$ is the mean of $X_{d}$.

1. **10th percentile**

The 10th percentile of $X_{d}$.

1. **90th percentile**

The 90th percentile of $X_{d}.$

1. **Quartile coefficient of dispersion (qcod)**

The quartile coefficient of dispersion is a robust alternative to the coefficient of variance.

$$qcod= \frac{P_{75}-P_{25}}{P_{75}+P_{25}}$$

where $P_{25}$ and $P_{75}$ are the 25th and 75th percentile of $X_{d}$, respectively.

**References**

1. Al-Kadi OS, ast, Watson D. Texture Analysis of Aggressive and Nonaggressive Lung Tumor CE CT Images. IEEE Transactions on Biomedical Engineering. 2008;55(7):1822-30.

2. Wahl RL, Jacene H, Kasamon Y, Lodge MA. From RECIST to PERCIST: Evolving Considerations for PET response criteria in solid tumors. Journal of nuclear medicine : official publication, Society of Nuclear Medicine. 2009;50 Suppl 1:122s-50s.
