# Supplementary material for: Challenges and caveats of a multi-center retrospective radiomics study: an example of early treatment response assessment for NSCLC patients using FDG-PET/CT radiomics
Source: PLoS One. 2019 Jun 3;14(6):e0217536. doi: 10.1371/journal.pone.0217536 (PMC6546238; doi:10.1371/journal.pone.0217536)
Supplement: S3 Text — Harrell’s concordance index with 95% confidence intervals for models trained on Dataset 2, 3, or 4 and validated on the remaining datasets. (DOCX) [file pone.0217536.s004.docx]

**Model Performance**

Values of Harrell’s concordance index with 95% confidence intervals for models trained on Dataset 2, 3 or 4 and validated on the remaining datasets. Values significant different from 0.5 are indicated in grey. A hyphen indicates that either all coefficients were forced to zero, or all predictions were equal to one, meaning that no linear combination of any subset of regressors was useful in predicting the outcomes. For Dataset 4, no subset of prognostic regressors was identified using the LASSO procedure for any of the images sets.

Training dataset: Dataset 2

|  | CT-scan1 | CT-scan2 | PET-scan1 | PET-scan2 | CT-rel | CT-abs | PET-rel | PET-abs |
| --- | --- | --- | --- | --- | --- | --- | --- | --- |
| **T-Dataset 2** | **0.76** | **-** | **0.84** | **0.71** | **-** | **-** | **0.84** | **0.86** |
| T-Lower bound | 0.66 | - | 0.75 | 0.64 | - | - | 0.79 | 0.82 |
| T-Upper bound | 0.87 | - | 0.93 | 0.78 | - | - | 0.89 | 0.91 |
| **V-Dataset 1** | **0.44** | **-** | **0.47** | **0.55** | **-** | **-** | **0.51** | **0.51** |
| V-Lower bound | 0.34 | - | 0.37 | 0.45 | - | - | 0.41 | 0.42 |
| V-Upper bound | 0.53 | - | 0.57 | 0.66 | - | - | 0.62 | 0.61 |
| **V-Dataset 3** | **0.53** | **-** | **0.51** | **0.40** | **-** | **-** | **0.54** | **0.59** |
| V-Lower bound | 0.41 | - | 0.36 | 0.28 | - | - | 0.40 | 0.47 |
| V-Upper bound | 0.64 | - | 0.65 | 0.52 | - | - | 0.69 | 0.71 |
| **V-Dataset 4** | **0.53** | **-** | **0.64** | **0.47** | **-** | **-** | **0.31** | **0.34** |
| V-Lower bound | 0.44 | - | 0.52 | 0.32 | - | - | 0.21 | 0.21 |
| V-Upper bound | 0.62 | - | 0.76 | 0.61 | - | - | 0.41 | 0.46 |

Training dataset: Dataset 3

|  | CT-scan1 | CT-scan2 | PT-scan1 | PT-scan2 | CT-rel | CT-abs | PT-rel | PT-abs |
| --- | --- | --- | --- | --- | --- | --- | --- | --- |
| **T-Dataset 3** | **-** | **-** | **-** | **-** | **0.71** | **0.74** | **-** | **-** |
| T-Lower bound | - | - | - | - | 0.59 | 0.64 | - | - |
| T-Upper bound | - | - | - | - | 0.84 | 0.83 | - | - |
| **V-Dataset 1** | **-** | **-** | **-** | **-** | **0.59** | **0.54** | **-** | **-** |
| V-Lower bound | - | - | - | - | 0.50 | 0.42 | - | - |
| V-Upper bound | - | - | - | - | 0.68 | 0.65 | - | - |
| **V-Dataset 2** | **-** | **-** | **-** | **-** | **0.60** | **0.55** | **-** | **-** |
| V-Lower bound | - | - | - | - | 0.48 | 0.44 | - | - |
| V-Upper bound | - | - | - | - | 0.72 | 0.67 | - | - |
| **V-Dataset 4** | **-** | **-** | **-** | **-** | **0.62** | **0.52** | **-** | **-** |
| V-Lower bound | - | - | - | - | 0.51 | 0.37 | - | - |
| V-Upper bound | - | - | - | - | 0.74 | 0.68 | - | - |

Training dataset: Dataset 4*

|  | CT-scan1 | CT-scan2 | PT-scan1 | PT-scan2 | CT-rel | CT-abs | PT-rel | PT-abs |
| --- | --- | --- | --- | --- | --- | --- | --- | --- |
| **T-Dataset 4** | **-** | **-** | **-** | **-** | **-** | **-** | **-** | **-** |
| T-Lower bound | - | - | - | - | - | - | - | - |
| T-Upper bound | - | - | - | - | - | - | - | - |
| **V-Dataset 1** | **-** | **-** | **-** | **-** | **-** | **-** | **-** | **-** |
| V-Lower bound | - | - | - | - | - | - | - | - |
| V-Upper bound | - | - | - | - | - | - | - | - |
| **V-Dataset 2** | **-** | **-** | **-** | **-** | **-** | **-** | **-** | **-** |
| V-Lower bound | - | - | - | - | - | - | - | - |
| V-Upper bound | - | - | - | - | - | - | - | - |
| **V-Dataset 3** | **-** | **-** | **-** | **-** | **-** | **-** | **-** | **-** |
| V-Lower bound | - | - | - | - | - | - | - | - |
| V-Upper bound | - | - | - | - | - | - | - | - |

***** No prognostic models could be identified using LASSO for any of the available image sets.
